# p53-Dependent Senescence in Mesenchymal Stem Cells under Chronic Normoxia Is Potentiated by Low-Dose *γ*-Irradiation

**DOI:** 10.1155/2016/6429853

**Published:** 2015-12-16

**Authors:** Ines Höfig, Yashodhara Ingawale, Michael J. Atkinson, Heidi Hertlein, Peter J. Nelson, Michael Rosemann

**Affiliations:** ^1^Institute of Radiation Biology, Helmholtz Center Munich, German Research Center for Environmental Health, Ingolstaedter Landstraße 1, 85764 Neuherberg, Germany; ^2^Chair of Radiation Biology, Technical University Munich, Ismaninger Straße 22, 81675 Munich, Germany; ^3^Research Group Clinical Biochemistry, Medical Clinic and Polyclinic IV, Medical Center of the University of Munich, Schillerstraße 42, 80336 Munich, Germany

## Abstract

Mesenchymal stem cells (MSCs) are a source of adult multipotent cells important in tissue regeneration. Murine MSCs are known to proliferate poorly* in vitro* under normoxia. The aim of this study is to analyze the interaction of nonphysiological high oxygen and low-dose *γ*-irradiation onto growth, senescence, and DNA damage. Tri-potent bone marrow-derived MSCs from p53 wildtype and p53−/− mice were cultured under either 21% or 2% O_2_. Long-term observations revealed a decreasing ability of wildtype mMSCs to proliferate and form colonies under extended culture in normoxia. This was accompanied by increased senescence under normoxia but not associated with telomere shortening. After low-dose *γ*-irradiation, the normoxic wildtype cells further increased the level of senescence. The number of radiation-induced *γ*H2AX DNA repair foci was higher in mMSCs kept under normoxia but not in p53−/− cells. P53-deficient MSCs additionally showed higher clonogeneity, lower senescence levels, and fewer *γ*H2AX repair foci per cell as compared to their p53 wildtype counterparts irrespective of oxygen levels. These results reveal that oxygen levels together with *γ*-irradiation and p53 status are interconnected factors modulating growth capacity of BM MSCs in long-term culture. These efforts help to better understand and optimize handling of MSCs prior to their therapeutic use.

## 1. Introduction

Mesenchymal stem cells (MSCs) are multipotent with a life-long proliferation capacity in the adult organism. Their potential to generate precursors for osteoblasts, adipocytes, fibroblasts, and chondroblasts* in vitro* [1, 2] has prompted the idea that they could also be a reservoir for the regeneration of connective tissue after injuries and fractures or during normal cell loss [3]. Because of their multilineage differentiation capacity and long-term proliferation potential, they have received interest as vehicles for the treatment of chronic degenerative diseases and acute tissue injuries [4]. The relative ease of harvesting facilitates autologous transplantation of MSCs into a diseased target organ, usually after the cells have been expanded* ex vivo* [5]. Some of their therapeutic benefits are also found to be conferred by anti-inflammatory and immunomodulatory factors secreted by the transplanted MSCs, rather than by the generation of viable cell progeny [6]. This may explain their reported efficiency as supplements in wound healing [7], bone marrow transplantation (to reduce graft-versus-host disease, GvHD) [8], and cardiac surgery [9].

At the time of writing, 529 clinical trials using MSCs are registered worldwide (https://www.clinicaltrials.gov/), with the majority intended to treat disorders of the CNS (12%), cardiac disease (10%), various types of autoimmune diseases (9%), joint and skeletal diseases (8%), metabolic disorders (7%), and GvHD (6%). Although the therapeutic procedures of autologous MSC transplantation are considered safe and more robust than allogenic transplantation protocols, the success of treatment in individual patients is variable [10]. To what extent transplanted MSCs proliferate and whether they are able to differentiate into fully functional cells capable of regenerating damaged tissue are usually not determined [11]. The mechanisms by which genetic, epigenetic, or environmental factors govern the efficiency of an MSC therapy are difficult to assess directly in patients but can be systematically studied in model organisms such as guinea pig, mice [12, 13], or minipig [14].

We are interested in the influence of genotoxic stress acting on MSCs both prior to and during* ex vivo* expansion, in particular by non-physiologically high oxygen levels and by ionizing radiation (IR). The sensitivity of eukaryotic cells to IR-induced cell killing depends on the presence of oxygen [15].* In vitro* studies of cancer cell lines show that hypoxia confers radioresistance, which is associated with a lower level of DNA lesions in the nucleus [16]. MSCs in their normal physiological context reside in hypoxic niches [17, 18] and might therefore be relatively protected from radical oxygen species (ROS), which are generated by ionizing radiation. In the case of haematopoietic stem cells derived from bone marrow, exposure to atmospheric oxygen has been shown to trigger EPHOSS (for extraphysiologic oxygen shock or stress) that is linked to activation of the p53 pathway and mitochondria-mediated apoptosis [19]. Although our own studies indicate that apoptosis is, unlike in haematopoietic cells, not a common mechanism in MSCs during aging or after cytogenetic stress, a p53-mediated DNA damage response (DDR) could also impair their stem cell potency by premature senescence or differentiation. Since murine MSCs growing* in vitro* depend on a hypoxic environment [20], they are a suitable model to investigate the mechanisms of oxygen induced cellular stress both alone and also in combination with a low-dose radiation exposure.

We have examined the growth capacity, clonogenic potential, senescence induction, accumulation of DNA damage, and rate of telomere attrition of mMSCs grown under two different oxygen concentrations. Since the first findings suggested senescence induction and involvement of accumulating DNA damage, we tested this assumption by low-dose *γ*-irradiation of mMSCs comparing p53 wild-type with p53−/− cells.

## 2. Materials and Methods

### 2.1. Cultivation of Primary Murine MSCs

Female FVB/N p53wt/wt and C57BL/6 p53−/− mice, 3 to 12 months old, were originally supplied by Charles River Laboratories (Sulzbach, Germany) and maintained in a breeding colony at the Helmholtz Center Munich under specific pathogen-free conditions. To collect bone marrow MSCs, mice were sacrificed by CO_2_ exposure, the hind limbs were aseptically dissected, and the femurs were cleaned from adherent tissues. One tip of each femur was removed and the bone marrow was collected by flushing with ice-cold PBS. After disaggregating larger clumps of bone marrow and allowing remaining cell clumps to sediment, the supernatant was centrifuged (5 min at 300 ×g), and the resulting cell pellet was washed again with PBS. Finally, bone marrow cells were plated at a cell density of 5,000,000 per 6-well plate in DMEM/F12 media containing 10% mesenchymal stem cell qualified FBS (Life Technologies, Carlsbad, CA). The cultures were kept in a humidified 5% CO_2_ incubator at 37°C under 21% (normoxia) or 2% (hypoxia) O_2_. After 4 h, 6 h, and twice a day for the following 3 days, supernatant containing nonadherent cells was aspirated and fresh complete medium was added. Adhering cells were grown for 7 days, detached for passaging with the StemPro Accutase Cell Dissociation Reagent (Life Technologies), and cell numbers were obtained using a Coulter Counter Analyzer (Beckman Coulter, Brea, CA). Growth medium was changed every 3.5 days and cells were passaged once a week.

### 2.2. *In Vitro* Induction of Lineage Differentiation

To verify the multilineage differentiation potential (Supplementary Figure 1 in Supplementary Material available online at http://dx.doi.org/10.1155/2016/6429853) the isolated mMSCs were plated at densities of 3,000 (osteogenic), 10,000 (adipogenic), or 80,000 (chondrogenic) cells per well in 96-well culture plates. For chondrogenic differentiation, cells were allowed to attach for 2 h before inducing the differentiation, using chondrogenic differentiation medium. In the case of osteogenic and adipogenic differentiation the cells were stimulated with appropriate differentiation medium only after 2 to 3 days. Remaining wells served as controls using normal MSC growth medium. All differentiation assays were performed under hypoxic conditions. Following lineage induction, cultures were stained and visualized on a Keyence BZ9000 microscope (Keyence, Neu-Isenburg, Germany).

#### 2.2.1. Alkaline Phosphatase Staining of Osteogenic Differentiation

Murine MSCs were stimulated for up to 2 weeks in StemPro osteocyte/chondrocyte differentiation basal medium supplemented with StemPro Osteogenesis Supplement (Life Technologies). After fixation of the cells with 4% paraformaldehyde, alkaline phosphatase activity was revealed by SigmaFast BCIP/NBT chromogen staining (Sigma Aldrich, St. Louis, MO) according to PromoCell's application note for “osteoblast differentiation and mineralization” (PromoCell GmbH, Heidelberg, Germany).

#### 2.2.2. Oil Red O Staining for Adipogenic Differentiation

Murine MSCs were stimulated for 2 weeks in StemPro Adipocyte differentiation basal medium supplemented with StemPro Adipogenesis Supplement (Life Technologies). After fixation of the cells with 4% paraformaldehyde, lipid vacuoles were stained for 45 min at room temperature with an Oil red O buffer solution (60% isopropanol with 3 mg/mL Oil red O (Sigma Aldrich), 40% H_2_O) and rinsed with water.

#### 2.2.3. Alcian Blue Staining for Chondrogenic Differentiation

Murine MSCs were stimulated for 3 weeks in StemPro Osteocyte/Chondrocyte differentiation basal medium supplemented with StemPro Chondrogenesis Supplement (Life Technologies). After fixation of the cells with 4% paraformaldehyde, proteoglycan aggrecan in the cartilage extracellular matrix was stained by an Alcian Blue 8GX dye (Sigma Aldrich) according to PromoCell's application note for “chondrogenic differentiation and analysis of MSC” (PromoCell).

### 2.3. Clonogenic Survival

For the measurement of clonogenic survival, between 500 and 5,000 mMSCs per 75 cm^2^ were seeded in cell culture flasks under either normoxia or hypoxia. After 10 to 14 days, the colony formation capacity was assayed after ethanol fixation and Giemsa (Merck, Darmstadt, Germany) staining. Plating efficiency in percentage was calculated by the following formula: (number of colonies formed/number of seeded cells) × 100.

### 2.4. Irradiation

Irradiation of mMSCs was performed with a Cs-137 irradiator (HWM D-2000, Siemens, Germany) at a dose rate of 0.5 Gy/min. For irradiation doses lower than 0.5 Gy, a shielded lead box was used with an attenuation factor of 10%. Doses were administered at room temperature and control cells were sham-irradiated. During the entire irradiation procedure (about 20 min) the cell culture vessels were sealed with parafilm to reduce gas exchange.

### 2.5. Senescence Analysis

For assessment of senescence associated *β*-galactosidase activity 7 days after irradiation, subconfluent mMSCs in 96-well culture plates were washed with PBS, fixed with 4% paraformaldehyde, again washed with PBS, and stained for 12 h at 37°C with an X-gal buffer solution (40 mM Na_2_HPO_4_, 150 mM NaCl, 2 mM MgCl_2_, 5 mM K_3_Fe(CN)_6_, 5 mM K_4_Fe(CN)_6_·3H_2_O, and 1 mg/mL X-gal, pH 6). Multicolored microscope images were taken with a Keyence BZ9000 microscope.

### 2.6. Analysis of p53 Stabilization and *γ*H2AX DNA Damage Repair Foci

Murine MSCs were seeded on glass slides and grown in either hypoxia or normoxia for at least 24 h. Following irradiation, the cells were fixed at the indicated time points (methanol for p53 or 4% paraformaldehyde for *γ*H2AX). Fixed cells were washed in PBS, treated with 0.05% Saponin (40 min for p53) or 0.2% Triton-X (5 min for *γ*H2AX) in PBS, washed in PBS, and blocked with 10 mg/mL BSA and 1.5 mg/mL glycine in PBS for 1 h at room temperature. Cells were incubated with primary antibodies against phosphorylated *γ*H2AX (Upstate Biotechnology, Lake Placid, NY) diluted in antibody dilution solution (DCS, Hamburg, Germany) or against p53 (R19, Santa Cruz Biotechnology, Dallas, TX) in PBS for at least 90 min. For visualization goat anti-mouse Cy3-conjugated secondary antibody (GE Healthcare GmbH, Freiburg, Germany) diluted in antibody dilution solution or HRP-conjugated donkey anti-goat secondary antibody (Santa Cruz Biotechnology) was used after washing slides in PBS (and blocking peroxidases for 15 min with 0.2% H_2_O_2_ for p53). For immunohistochemical staining of p53, SIGMAFAST DAB (3,3′-diaminobenzidine tetra hydrochloride, Sigma Aldrich) was added overnight. After a final PBS washing step slides were mounted in DAPI-containing mounting medium and analyzed with a Keyence BZ9000 microscope. The Keyence software counted nuclear foci in at least 50 cells. Appropriate negative controls were obtained by staining a replicate slide with the secondary antibody only.

### 2.7. Telomere Length Determination

Quantitative PCR of genomic DNA extracted from mMSCs was done to measure telomeric length [21]. For this purpose, qRT-PCR was set up using (TTAGGG)n primers detecting the telomere sequence and primers for a genomic microsatellite marker (D14Mit192) for genomic DNA normalization in two separate reactions. Quantitative real-time amplification was done on a StepOne^+^ device (Life Technologies), using the “Power Sybr-Green” master mix (including Rox-standard), 2 pmol of each primer, and 10 ng genomic DNA in a 20 *μ*L reaction volume. Relative telomeric length was calculated by the delta-delta CT method, with pooled newborn mouse skin DNA as a calibrator (set as 100 arbitrary units, AU).

### 2.8. Triplex Assay to Quantify Viability, Cytotoxicity, and Apoptosis Induction

Murine MSCs were seeded on 96-well plates, irradiated, and incubated in either hypoxia or normoxia for 7 days. Viability, cytotoxicity, and apoptosis were assessed in one well using the ApoTox-Glo Triplex assay (Promega, Madison, WI). In a first step, the cell-permeable substrate glycylphenylalanyl-aminofluoro-coumarin (GF-AFC) is cleaved by live-cell protease activity to generate a fluorescent signal (400 nm excitation, 505 nm emission) proportional to the number of living cells. In parallel the nonpermeable substrate bis-alanylalanyl-phenylalanyl-rhodamine 110 (bis-AAF-R110; 485 nm excitation, 520 nm emission) is used to measure dead-cell protease activity, which is released from cells that have lost membrane integrity. In a second step, the addition of CaspaseGlo 3/7 reagent results in cell lysis, followed by caspase cleavage of the substrate and generation of luminescent signal produced by luciferase. Both fluorescence and luminescence signals were analyzed in a microplate reader (Infinite 200, Tecan, Männedorf, Switzerland). Positive controls for necrosis had cells exposed for 6 h to 100 *μ*M ionomycin, and positive controls for apoptosis had cells exposed to 100 *μ*M staurosporine for 6 h (both Sigma Aldrich), incubated under hypoxia.

### 2.9. Statistical Analysis

All experiments were performed with at least duplicate technical and biological replicates. Mean ± standard errors of the mean (SEM) values are depicted unless stated otherwise. Results were statistically evaluated with indicated tests by the statistic software SigmaPlot (Systat Software Inc., San Jose, CA) and SPSS version 22 (IBM Corp, Armonk, NY). Statistical significance was accepted at the *p* < 0.05 level.

## 3. Results

### 3.1. Oxygen Concentration Affects Long-Term* In Vitro* Proliferative Potential of Murine MSCs

**Figure 1:**
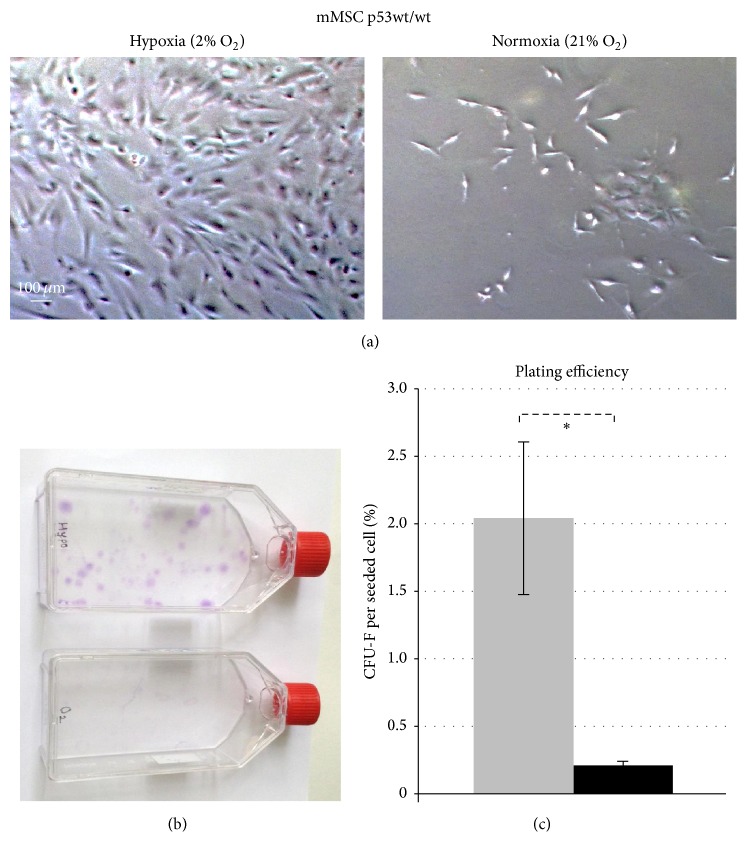
Effects of oxygen levels on long-term* in vitro* culturing and colony forming capacity of p53 wild-type mMSCs. (a) Proliferation and morphology of mMSCs p53wt/wt cultured in hypoxic conditions (2% O_2_, left) and in normoxic conditions (21% O_2_, right). Representative images show mMSCs cultured for 32 days. (b) Colony forming ability of mMSCs in hypoxic (upper) and normoxic (lower) atmospheres 14 days after plating 5,000 cells in a 75 cm^2^ flask. (c) Plating efficiency calculated as mean values of fibroblast colony forming units (CFU-F) per plated cell ± standard error of the mean (*n* = 4) in hypoxic (grey bar) and normoxic (black bar) conditions.

In line with previously published studies [22], we observed increased proliferation of mMSCs in a 2% O_2_ (hypoxic) environment as compared to the 21% O_2_ (normoxic) environment. As the time in culture progressed, the total cell numbers in hypoxic cultures were significantly higher as compared to those in normoxic culture conditions. Representative images show long-term cultured hypoxic and normoxic mMSCs on day 32 after isolation (Figure 1(a)).

### 3.2. Normoxic Culture Condition Reduces Colony Forming Ability of mMSCs

The reduced cellularity of normoxia grown mMSC cultures was due to a reduced ability to form colonies. Thus, clonogenic assays of mMSCs revealed a tenfold higher number of colonies (2.04%) under hypoxia, as compared to growth under normal oxygen conditions (0.21%) (Figures 1(b) and 1(c)) (*p* = 0.03, one-tailed paired Student's *t*-test). These results prove that the clonogenicity of mMSCs is impaired in normoxic cultures.

### 3.3. Normoxia Increases the Sensitivity of mMSCs towards Radiation-Induced Senescence

**Figure 2:**
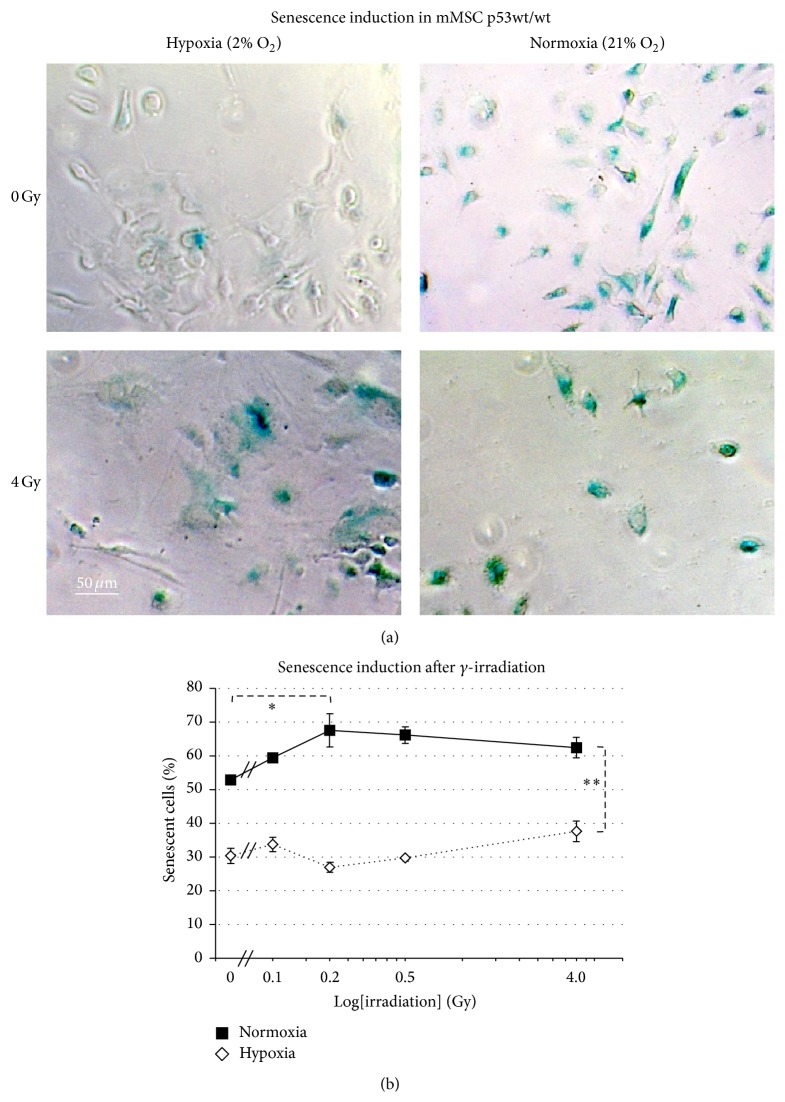
Effects of oxygen levels on sensitivity of p53 wild-type mMSCs for radiation-induced senescence. (a) Green SA *β*-gal positive mMSCs in sham-irradiated (0 Gy) hypoxic (upper left) and in normoxic cultures (upper right) and 7 days after exposure to 4 Gy *γ*-irradiation (hypoxia, lower left, and normoxia, lower right). (b) Percentage of cells undergoing senescence in hypoxic and normoxic conditions after increasing doses of *γ*-irradiation depicted as mean values ± standard error of the mean (4 biological replicates), radiation dose on logarithmic scale.

We have not seen a relevant increase of necrotic or apoptotic cells using a multiplex assay with fluorescent GF-AFC/bis-AAF-R100 and luminescent caspase-3/-7 substrates (Supplementary Figure 2), neither after irradiation nor by normoxia. So we hypothesized that senescence is the key mechanism of decreased proliferative and loss of colony forming ability of mMSCs in cultures with ambient oxygen concentration (21% O_2_). To investigate the possible role of oxygen on senescence induction, subconfluent mMSC cultures under hypoxic and normoxic conditions were stained for senescence-associated *β*-galactosidase (SA *β*-gal) activity. In sham-irradiated cells that were transferred to normoxic conditions, we observed a strong increase to 54 ± 1.2% senescent cells, as compared to 31 ± 2.8% in cells kept under hypoxic conditions for the same time (Figure 2(a)).

Ionizing radiation (IR) is known to generate oxidative stress in cells [23]. Therefore, we exposed mMSCs maintained in hypoxic and normoxic conditions to increasing doses of *γ*-irradiation (0.1 Gy, 0.2 Gy, 0.5 Gy, and 4 Gy). Cells irradiated with a dose as high as 4 Gy exhibited a change in the MSCs' typical spindle cell morphology, which might indicate cellular stress. There was also a much reduced cell growth of MSCs following 4 Gy irradiation, as measured by lower cell numbers after 7 days under both oxygen concentrations (Figure 2(a)). The combination of higher oxygen and IR significantly increased senescence in mMSCs (*p* = 0.003, ANOVA). Irradiation could independently cause mMSCs to undergo senescence (*p* = 0.031, ANOVA). Ambient oxygen concentration was also found to be a crucial factor responsible for increasing senescence in mMSCs (*p* = 3.2 × 10^−18^, ANOVA). All together this suggests that normoxia increases the sensitivity of mMSCs towards radiation-induced senescence.

### 3.4. Murine MSCs Grown in Normoxia Have Higher Basal Levels of DNA Damage and Show a Greater Increase after *γ*-Irradiation

**Figure 3:**
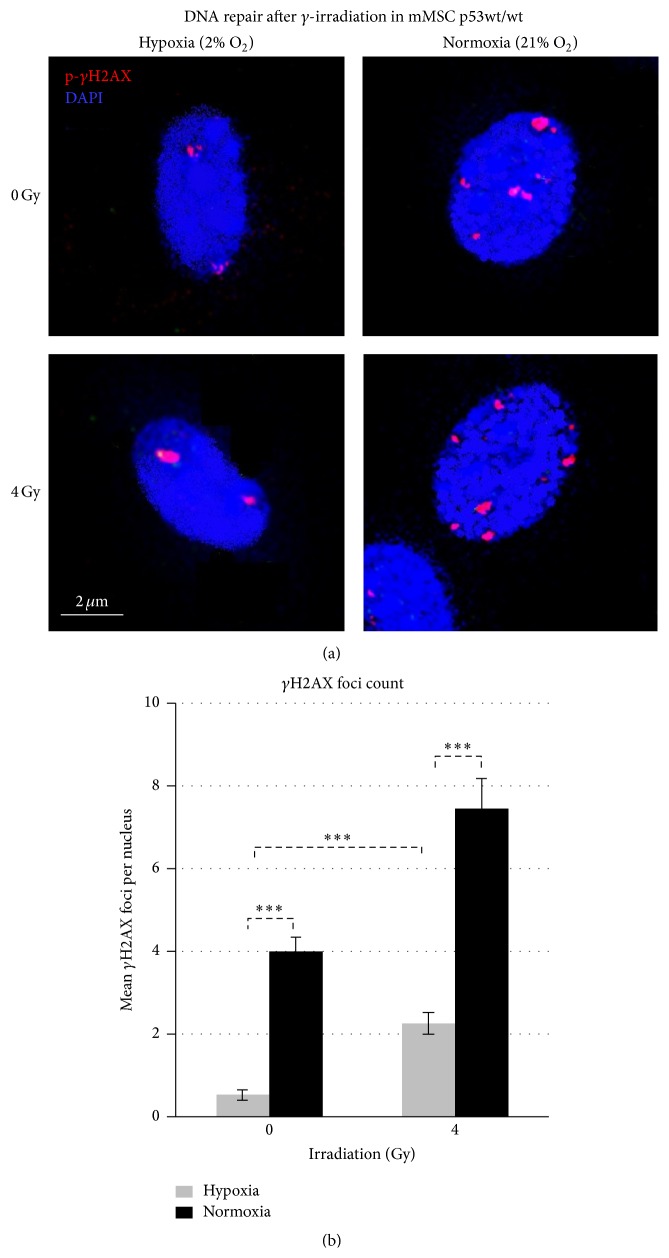
Effects of oxygen levels on the number of repair foci in sham-irradiated and 4 Gy irradiated p53wt/wt mMSCs. (a) Sham-irradiated (0 Gy) *γ*H2AX foci formation under normoxic cultures (right upper image) and hypoxic cultures (left upper image) and additionally 90 min after exposure to 4 Gy *γ*-irradiation (lower images). (b) Quantification of *γ*H2AX foci in 50 p53wt/wt mMSC nuclei depicted as mean values ± standard error of the mean (3 biological replicates).

In sham-irradiated mMSCs under hypoxia, most of the cells showed an absence of *γ*H2AX foci, with a mean value of 0.5 (range from 0 to 2) foci per nucleus (Figures 3(a) and 3(b)). The number and the size of the *γ*H2AX foci were significantly increased when cells were grown under normoxic conditions, reaching mean values of 4.0 (range from 2 to 6) foci per cell.

4 Gy irradiated mMSCs cultured under hypoxia increased the number of foci per cell to a mean value of 2.3 (range from 0 to 4) foci per nucleus (Figures 3(a) and 3(b)), indicating ongoing repair. 4 Gy irradiated mMSCs cultured under normoxia showed massive appearance of *γ*H2AX foci with a mean value of 7.5 per nucleus (range from 4 to more than 20 foci in some cells). These experiments indicate that normoxia increases the sensitivity of mMSCs towards DNA lesions when *γ*-irradiation is applied.

### 3.5. No Effect of Normoxia on mMSC Telomere Shortening

**Figure 4:**
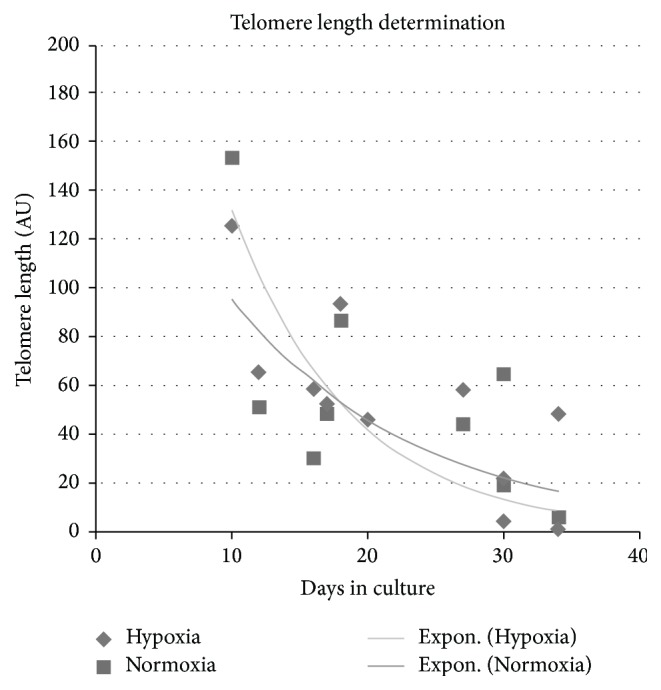
Effects of oxygen levels on telomeric length. Graph shows quantitative genomic PCR telomeric length determination performed with p53wt/wt mMSCs under hypoxic and normoxic conditions and with a pool of newborn mouse skin DNA as calibrator (set to 100 arbitrary units, AU). Fitted lines are exponential regression curves, and the differences are not significant.

DNA damage signaling can also be triggered by dysfunctional, eroding telomeres [24]. Therefore the relative telomere length of mMSCs growing under hypoxia and under normoxia was measured for 3 weeks in culture (Figure 4). At the beginning of the culture period, the telomere length in mMSCs was slightly longer (123 AU to 155 AU) than in the reference cells (new-born skin fibroblasts, set as 100 AU), but this declined during* in vitro* expansion. Within 24 days, telomere length had decreased to 18 ± 7 AU (normoxia) or 5 ± 5 AU (hypoxia), equivalent to a half-life time of about 7.8 days. Exponential fitting of the time course of telomere shortening did not yield significant differences in the kinetic parameter between the hypoxic and the normoxic curve.

### 3.6. Long-Term* In Vitro* Proliferative Potential of mMSCs Is Not Affected by Oxygen Conditions in the Absence of p53

**Figure 5:**
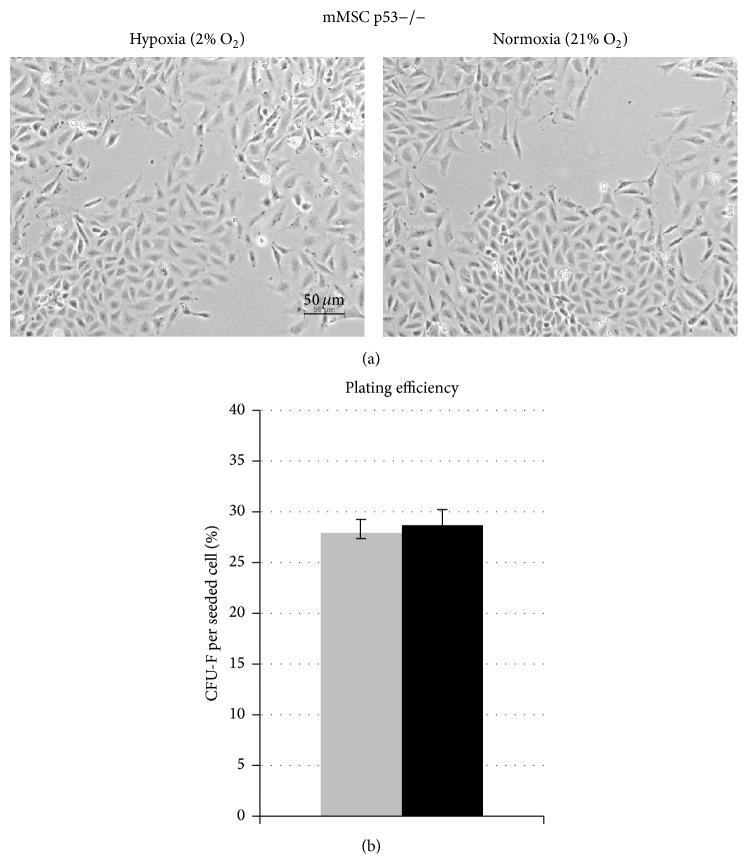
Impact of p53 status on oxygen effect of long-term* in vitro* culturing and colony formation capacity of mMSCs. (a) Morphology of mMSCs p53−/− cultured in hypoxic conditions (left image) and in normoxic conditions (right image). Representative images show mMSCs p53−/− cultured for 20 days. (b) Colony forming ability of mMSCs p53−/− after 14 days in hypoxic (grey bar) and normoxic (black bar) cultures calculated as mean values of fibroblast colony-forming units (CFU-F) per plated cell (3 biological replicates).

We found evidence of higher levels of senescence and DNA strand breaks to be present in mMSCs when kept under normoxia (21% O_2_). The transcription factor p53 regulates a variety of target genes affecting several cellular pathways, all involved in establishment of both senescence and DNA damage response [25, 26]. More prominent stabilization of p53 after irradiation was observed as a feature of mMSCs incubated under normoxia than under hypoxia (Supplementary Figure 3). Using MSCs from p53−/− mice, we found that* in vitro* proliferation was unaffected by normal oxygen concentration (Figure 5(a)). The plating efficiency as a measure of clonogenic potential of these cells was higher (28% ± 3%) than in the p53wt/wt cells (2% ± 0.6%) and it was unaffected by the oxygen concentration (Supplementary Figure 4 and Figure 5(b)).

### 3.7. Senescence and *γ*H2AX Repair Foci Formation Are Reduced in p53−/− Irrespective of Oxygen Levels

**Figure 6:**
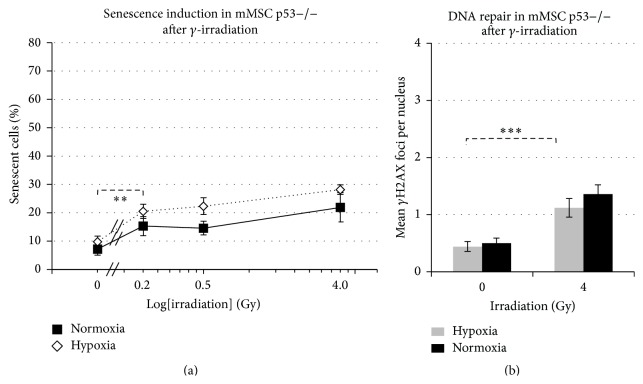
Impact of p53 status on senescence induction and number of DNA repair foci. (a) Graph shows percentage of p53−/− mMSCs undergoing senescence in hypoxic and normoxic conditions after exposure to *γ*-irradiation (sham-irradiated = 0 Gy, 3 biological replicates), radiation dose on logarithmic scale. (b) Quantification of p-*γ*H2AX foci in 50 p53−/− mMSC nuclei depicted as mean values ± standard error of the mean (3 biological replicates).

In p53-deficient mMSCs, the spontaneous frequency of senescence was significantly lower as compared to senescence in p53wt/wt mMSCs, both under hypoxic growth conditions (9.8% ± 1.5%), as well as in cells kept in normoxia (7.2% ± 1.8%) (Figure 6(a)). The difference of 2.6% (±3.3%) in the senescence level between hypoxic and normoxic p53−/− mMSCs remained unaltered after additional *γ*-irradiation (0.2 Gy, 0.5 Gy and 4 Gy), despite the frequency itself being increased in a similar fashion as in normoxic p53wt/wt cells (Figure 2(b)).

The number of radiation-induced DNA double strand breaks (*γ*H2AX repair foci) under both hypoxic and normoxic conditions (0.4 foci per cell were similar to the results of p53wt/wt cells under hypoxia) is in line with our previous observations (Figure 6(b)). Basal levels of *γ*H2AX repair foci after sham-irradiation (0 Gy) were in the range of hypoxic p53 wild-type mMSCs, with mean values of 0.4 ± 0.08 and 0.5 ± 0.08 foci per nucleus in hypoxia and normoxic conditions, respectively. After *γ*-irradiation of 4 Gy, the number of *γ*H2AX repair foci after 90 min increased significantly to 1.1 (normoxia) or 1.4 (hypoxia) foci per nucleus, representing highly effective ongoing DNA repair compared to p53 wild-type mMSCs irrespective of oxygen conditions applied. These results show that p53 is a key factor in modulating both senescence induction and DNA repair efficiencies in mMSCs.

## 4. Discussion

Mesenchymal stem cells in their normal physiological state reside in a stem cell niche with low-oxygen pressure [17, 18]. Since they have a lower metabolic rate than the committed precursor cells and differentiated cells derived from them [27, 28], they are less dependent on high oxygen supply. Considering that their main function is the long-term regeneration of precursor cells of different lineages and hence the maintenance of a stable genome, a reduction of radical oxygen species (ROS) in a tissue environment with lower oxygen pressure and low metabolic activity could be beneficial. This protective hypoxic environment is compromised when MSCs are isolated and expanded* ex vivo* but also by exposure to any kind of ionizing radiation (IR)* in vivo* or* in vitro*. IR has the capacity to generate ROS- and OH-radicals from intracellular water, thereby bypassing the otherwise protective hypoxic stem cell niche [29].

We have shown that growth under ambient oxygen levels (i.e., 21%) compromises MSC proliferation when compared to growth under hypoxia (i.e., 2%). This is in line with observations by others, who suggested using reduced oxygen cell culture systems [22, 30]. In a therapy model, it has been shown that transient maintenance of human MSCs in hypoxia prior to autologous therapy increased their efficacy in the treatment of idiopathic pulmonary fibrosis, and that this was accompanied by enhanced survival of the grafted mMSCs [31].

We have shown here that the impairment of MSC proliferation under normoxia was associated with an increase in cellular senescence. Senescence would not only preclude further stem cell expansion but also would hamper the differentiation capacity of the already existing cells [32]. Senescence can be triggered by various cellular signals: shortened telomeres that bring dividing cells close to the Hayflick limit [33], accumulated DNA damage [34], or activated oncogenes [35]. Measuring the average telomere length in the proliferating MSCs over several weeks in culture showed a clear reduction with time but no difference between cells grown under normoxia or low oxygen. We can therefore exclude that telomere shortening is involved in the oxygen sensitivity of MSCs.

There was, however, a striking increase in the level of DNA damage as detected by *γ*H2AX repair foci in cells that stopped growing after two weeks in normoxia. We tested the assumption that DNA lesions are causing senescence in MSCs by subjecting cells in hypoxia and in normoxia to various doses of Cs-137 *γ*-irradiation. With 500 mGy, we observed a significant increase in the frequency of senescent cells grown in normoxia compared to cells grown under hypoxic conditions. A radiation dose of this range could be delivered to long-living stem cells by 25 repeated computer tomography exposures (20 mGy each) and should warrant* ex vivo* expansion of MSCs collected from parts of the human body that might have been exposed to repeated radiation doses in the past. MSCs kept at 2% low oxygen exhibit signs of senescence only in 31% of cells as compared to 53% in normoxic cells. MSCs grown under reduced oxygen not only exhibit a lower percentage of senescent cells but also responded much less severe to *γ*-irradiation. Up to a dose of 500 mGy, the relative number of senescent cells did not increase above the control level, and we had to expose the cells with 4000 mGy to see a slight increase in senescence to 39%. This shows that the detrimental effects of ionizing radiation on MSCs proliferation are dependent on a non-physiologically high oxygen atmosphere (EPHOSS [19]) during* ex vivo* growth, which should be avoided while explanting as well as* ex vivo* expanding of MSCs in particular from patients who might have accumulated radiation-induced DNA damage in their MSCs prior to an autologous therapy.

*In vitro* expanded hMSCs treated with ROS-inducing chemical were found to exhibit an increased number of 53BP1-stained DNA repair foci, which in part could be rescued by adding an antioxidant [36]. This shows that the potential for induction of damage to the genomic DNA is greater under high oxygen levels but does not exclude other cellular targets (such as membranes or mitochondria) as potential mediators of the effect.

The suggestion that DNA damage induced by non-physiologically high oxygen and/or ionizing radiation can act synergistically in causing premature cell senescence was strengthened by the observation that MSCs with a homozygous deletion in the p53 gene (i.e. the main signal transducer for DNA damage response) grow considerably faster than wild-type MSCs, both under normoxia and under hypoxia. It is interesting to note that p53−/− mice do not show any defect in the growth or regeneration of mesenchymal tissue, rather an increased rate of tumors derived from cells of all germ layers [37]. This suggests that p53 is dispensable for the normal physiological function of MSCs. On the other hand, it was shown that hMSCs expanded for a prolonged time in* in vitro* hypoxia exhibit a change in their gene expression profile, in particular for three transcription factors (c-MYC, p53, and HIF1) that are frequently amplified, mutated, or overexpressed in cancer [38] and that this led to a higher transformation frequency after exposure of the cells to NiSO_4_. One suggestion from this observation was that genomic instability could be the result of forced proliferation and hypoxia. Our data show, however, that MSCs under hypoxia rather exhibit less spontaneous DNA strand breaks, therefore excluding the possibility that hypoxia per se is genotoxic. But it might be that premature senescence triggered by non-physiologically high oxygen indeed prevents a malignant progression, albeit on the expense of reduced cell proliferation.

## 5. Conclusions

We have shown that impaired growth of MSCs under a normoxic atmosphere* ex vivo* is associated with accumulated DNA damage but not with additional telomere attrition. This impaired growth causes not only a reduced cell number but also, due to increased senescence, a loss of stem cell potency. The observation that radiation-induced DNA damage and high oxygen atmosphere seem to act synergistically advices caution not to explant MSCs from regions of the body that might have been radiation-exposed earlier in life, as for diagnostic X-ray imaging or therapeutic purposes. It might even be an option to store MSCs from tissues easily available early in life, thus sparing them from excess radiation exposure and having them available as a repository for autologous organ regeneration later in life.

## Supplementary Material

Supplementary material contains an overview over the tri-lineage differentiation potential of *in vitro* cultured p53 wild-type mMSCs to form osteocytes, chondrocytes and adipocytes (S1). Multiplex quantification of viability (S2a), cytotoxicity (S2b) and apoptosis induction (S2c) of mMSCs after γ-irradiation under normoxia and hypoxia is shown in the next figure including apoptosis- and necrosis-inducing agents as positive controls. Supplementary Figure S3 describes the effects of oxygen levels on p53 protein stabilization measured by immunohistochemistry in sham-irradiated and 4 Gy irradiated mMSCs, p53-deficient mMSCs served as negative controls. Colony formation in p53wt/wt (a) and p53-/- mMSCs (b) under hypoxic and normoxic conditions as basis for CFU-F calculation is shown in supplementary figure S4.
