# Supplementary material for: p53-Dependent Senescence in Mesenchymal Stem Cells under Chronic Normoxia Is Potentiated by Low-Dose γ-Irradiation
Source: Stem Cells Int. 2015 Dec 16;2016:6429853. doi: 10.1155/2016/6429853 (PMC4695678; doi:10.1155/2016/6429853)
Supplement: Supplementary file 1 — Supplementary material contains an overview over the tri-lineage differentiation potential of in vitro cultured p53 wild-type mMSCs to form osteocytes, chondrocytes and adipocytes (S1). Multiplex quantification of viability (S2a), cytotoxicity (S2b) and apoptosis induction (S2c) of mMSCs after γ-irradiation under normoxia and hypoxia is shown in the next figure including apoptosis- and necrosis-inducing agents as positive controls. Supplementary Figure S3 describes the effects of oxygen levels on p53 protein stabilization measured by immunohistochemistry in sham-irradiated and 4 Gy irradiated mMSCs, p53-deficient mMSCs served as negative controls. Colony formation in p53wt/wt (a) and p53-/- mMSCs (b) under hypoxic and normoxic conditions as basis for CFU-F calculation is shown in supplementary figure S4. [file 6429853.f1.zip › Suppl_Figure_1.doc]

**Supplementary Figure 1: Tri-lineage differentiation potential of *in vitro* cultured mMSCs p53wt/wt (hypoxia, 2% O_2_).** Osteogenic differentiation (left column): Murine MSCs induced to undergo osteogenic differentiation (upper row image) and negative control cells (lower row image) stained for alkaline phosphatase activity. Chondrogenic differentiation (middle column): Alcian blue staining of mMSCs under induced chondrogenic differentiation (chondrogenic pellets, upper row image) and negative control cells (lower row image). Adipogenic differentiation (right column): Lipid vacuoles are stained with Oil red O in mMSCs induced to undergo adipogenic differentiation (upper row image) and in negative control cells (lower row image).

**Osteogenic**

**Alkaline phosphatase^+^**

**Adipogenic**

**Lipid vacuoles^+^**

**Differentiation of mMSCs in hypoxia (2% O_2_)**

**Chondrogenic**

**Proteoglycan aggrecan^+^**

**Giemsa stain added**

**100 µm**

**25 µm**

**25 µm**
