# Supplementary material for: p53-Dependent Senescence in Mesenchymal Stem Cells under Chronic Normoxia Is Potentiated by Low-Dose γ-Irradiation
Source: Stem Cells Int. 2015 Dec 16;2016:6429853. doi: 10.1155/2016/6429853 (PMC4695678; doi:10.1155/2016/6429853)
Supplement: Supplementary file 1 — Supplementary material contains an overview over the tri-lineage differentiation potential of in vitro cultured p53 wild-type mMSCs to form osteocytes, chondrocytes and adipocytes (S1). Multiplex quantification of viability (S2a), cytotoxicity (S2b) and apoptosis induction (S2c) of mMSCs after γ-irradiation under normoxia and hypoxia is shown in the next figure including apoptosis- and necrosis-inducing agents as positive controls. Supplementary Figure S3 describes the effects of oxygen levels on p53 protein stabilization measured by immunohistochemistry in sham-irradiated and 4 Gy irradiated mMSCs, p53-deficient mMSCs served as negative controls. Colony formation in p53wt/wt (a) and p53-/- mMSCs (b) under hypoxic and normoxic conditions as basis for CFU-F calculation is shown in supplementary figure S4. [file 6429853.f1.zip › Suppl_Figure_3.doc]

**mMSCs p53-/-**

**mMSCs p53wt/wt**

**mMSCs p53wt/wt**

**Hypoxia (2% O_2_)**

**Normoxia (21% O_2_)**

**Supplementary Figure 3: Effects of oxygen levels on p53 protein stabilization in sham-irradiated and 4 Gy irradiated mMSCs.** Representative images of immunohistochemical analysis (DAB) of p53 protein levels in sham-irradiated (0 Gy) p53 wt mMSCs 72 h incubated under normoxic culture conditions (right upper image, 5 stained nuclei per 100 nuclei) and hypoxic cultures (middle upper image, 4 stained nuclei per 100 nuclei). 60 min after exposure to 4 Gy γ-irradiation, p53 levels increase under normoxic conditions (left lower image, 84 stained nuclei per 100 nuclei) and hypoxic conditions (middle lower image, 57 stained nuclei per 100 nuclei). P53-negative mMSCs were analysed as negative controls (upper and lower right images) (scale bar: 50 µm, 2 biological replicates).
