# Supplementary material for: p53-Dependent Senescence in Mesenchymal Stem Cells under Chronic Normoxia Is Potentiated by Low-Dose γ-Irradiation
Source: Stem Cells Int. 2015 Dec 16;2016:6429853. doi: 10.1155/2016/6429853 (PMC4695678; doi:10.1155/2016/6429853)
Supplement: Supplementary file 1 — Supplementary material contains an overview over the tri-lineage differentiation potential of in vitro cultured p53 wild-type mMSCs to form osteocytes, chondrocytes and adipocytes (S1). Multiplex quantification of viability (S2a), cytotoxicity (S2b) and apoptosis induction (S2c) of mMSCs after γ-irradiation under normoxia and hypoxia is shown in the next figure including apoptosis- and necrosis-inducing agents as positive controls. Supplementary Figure S3 describes the effects of oxygen levels on p53 protein stabilization measured by immunohistochemistry in sham-irradiated and 4 Gy irradiated mMSCs, p53-deficient mMSCs served as negative controls. Colony formation in p53wt/wt (a) and p53-/- mMSCs (b) under hypoxic and normoxic conditions as basis for CFU-F calculation is shown in supplementary figure S4. [file 6429853.f1.zip › Suppl_Figure_4.doc]

**Hypoxia (2% O_2_)**

**Normoxia (21% O_2_)**

**mMSC p53-/-**

**Hypoxia (2% O_2_)**

**Normoxia (21% O_2_)**

**mMSC p53wt/wt**

**A**

**B**

**Colony forming capacity**

**Supplementary Figure 4: Colony formation in p53wt/wt and p53-/- mMSCs under hypoxic and normoxic conditions.** Images show representative flasks (75 cm^2^) with Giemsa-stained colonies from 5,000 p53wt/wt (A) and 500 mMSCs p53-/- (B) as basis for counting and calculating plating efficiencies.
